# Long term outcome after toxic nodular goitre

**DOI:** 10.1186/s13044-022-00138-0

**Published:** 2022-11-01

**Authors:** Gabriel Sjölin, Torquil Watt, Kristina Byström, Jan Calissendorff, Per Karkov Cramon, Helena Filipsson Nyström, Bengt Hallengren, Mats Holmberg, Selwan Khamisi, Mikael Lantz, Tereza Planck, Ove Törring, Göran Wallin

**Affiliations:** 1 Faculty of Medicine and Health, Örebro University Hospital, Örebro, Sweden; 2 Dept. of Surgery, Örebro University and University Hospital, 701 85 Örebro, Sweden; 3 Department of Medical Endocrinology Rigshospitalet, Copenhagen, Denmark; 4 Internal Medicine Herlev Gentofte Hospital, Copenhagen University Hospital, Copenhagen, Denmark; 5 Dept. of Medicine, Örebro University and University Hospital, Örebro, Sweden; 6 Department of Molecular Medicine and Surgery, Karolinska Institutet, Stockholm, Sweden; 7 Dept. of Endocrinology, Metabolism and Diabetes, Karolinska University Hospital, Stockholm, Sweden; 8 Institute of Medicine, Sahlgrenska Academy, University of Gothenburg, Göteborg, Sweden; 9 Dept. of Endocrinology, Sahlgrenska University Hospital, Göteborg, Sweden; 10 Wallenberg Center for Molecular and Translational Medicine, Göteborg, Sweden; 11 Dept. of Endocrinology, Skåne University Hospital, Malmö, Sweden; 12 Dept. of Clinical Sciences, Lund University, Lund, Sweden; 13 ANOVA, Karolinska University Hospital, Stockholm, Sweden; 14 Dept. of Endocrinology, Uppsala University Hospital, Uppsala, Sweden; 15 Dept. of Medical Sciences, Uppsala University, Uppsala, Sweden; 16 Institution for Clinical Science and Education, Karolinska Institutet, Stockholm, Sweden

**Keywords:** Hyperthyroidism, Toxic nodular goitre, Anti-thyroid drugs, Radioactive iodine, Thyroidectomy, Long-term follow-up, Recurrence, Cure, Quality of life

## Abstract

**Background:**

The purpose of treating toxic nodular goitre (TNG) is to reverse hyperthyroidism, prevent recurrent disease, relieve symptoms and preserve thyroid function. Treatment efficacies and long-term outcomes of antithyroid drugs (ATD), radioactive iodine (RAI) or surgery vary in the literature. Symptoms often persist for a long time following euthyroidism, and previous studies have demonstrated long-term cognitive and quality of life (QoL) impairments. We report the outcome of treatment, rate of cure (euthyroidism and hypothyroidism), and QoL in an unselected TNG cohort.

**Methods:**

TNG patients (*n* = 638) de novo diagnosed between 2003–2005 were invited to engage in a 6–10-year follow-up study. 237 patients responded to questionnaires about therapies, demographics, comorbidities, and quality of life (ThyPRO). Patients received ATD, RAI, or surgery according clinical guidelines.

**Results:**

The fraction of patients cured with one RAI treatment was 89%, and 93% in patients treated with surgery. The rate of levothyroxine supplementation for RAI and surgery, at the end of the study period, was 58% respectively 64%. Approximately 5% of the patients needed three or more RAI treatments to be cured. The patients had worse thyroid-related QoL scores, in a broad spectrum, than the general population.

**Conclusion:**

One advantage of treating TNG with RAI over surgery might be lost due to the seemingly similar incidence of hypothyroidism. The need for up to five treatments is rarely described and indicates that the treatment of TNG can be more complex than expected. This circumstance and the long-term QoL impairments are reminders of the chronic nature of hyperthyroidism from TNG.

## Introduction

Toxic nodular goitre (TNG), defined as both toxic multinodular goitre and toxic adenoma, affects 6.5/100,000 inhabitants/year in Sweden, where there is an incidence of hyperthyroidism of 27.6/100,000 inhabitants/year [1]. Among the hyperthyroid patients, TNG is more prevalent in older ages, often has a milder course and is subclinical in the majority of cases [2, 3]. However, older patients with TNG may be frail and more prone to parallel morbidity during the phase of hyperthyroidism than younger patients with Graves’ disease (GD) [4].

There are only a few treatment options for TNG, and the two main therapies are radioactive iodine (RAI) and surgery [5]. Antithyroid drugs (ATD) can be a possible treatment option when the other treatments are contraindicated [5].

Earlier studies show a relatively low (0─36%) frequency of hypothyroidism after RAI treatment [6–8], but a more recent, long-term follow-up study suggests a much higher frequency (73%) [9]. Surgery, which is often performed as a lobectomy or total thyroidectomy, carries a risk for damage to the recurrent laryngeal nerves and the induction of hypoparathyroidism [10]. On the other hand, surgery has a recurrence rate near zero, while 15–25% of patients receiving RAI experience recurrence, resulting in the need for additional treatment [6–9, 11].

TNG is accompanied by increased long-term morbidity and mortality [12]. In addition, long-term cognitive deficits and impairment of quality of life (QoL) [4, 12–15] are seen, with symptoms often remaining for a long time after euthyroidism is achieved [16, 17]. It has previously been shown that patients with active TNG have a lower QoL than the general population [18], but treatment of TNG improves the QoL [16, 18, 19]. However, how different treatments influence QoL is still not established, even though some studies indicate that surgery, on its own, can restore the QoL [19]. Therefore, while treatment results in a higher QoL than before treatment, the overall impairment persists at least 6 months after treatment [18] or longer [16, 17].

The outcomes of TNG may thereby be measured as its long-term impact on QoL, socioeconomic consequences from inability to work, increased morbidity and mortality, and the thyroid treatments requiring life-long levothyroxine supplementation.

There are few numerically large, long-term follow-up studies concerning TNG [7], in regard to not only treatment but also QoL, and there are only a limited number of studies that include all treatment options and, to the authors’ knowledge, none with follow-up longer than a year related to QoL. Thus, the long-term effects of TNG are unknown regarding both QoL and the treatment outcomes.

- (a) to explore the efficacy of each therapeutic modality on disease remission and normalization of thyroid function and, subsequently, the utilization of each therapy during the entire period along with the patient’s satisfaction with each treatment modality.
- (b) to assess the long-term QoL impact of TNG in patients treated in a routine clinical setting, both in comparison with the general population as well as between different treatment modalities.

We pursued these aims in a subnational incidence cohort from 2003–05 comprised of 638 patients [1].

## Materials and methods

### Study design and subjects

**Fig. 1:**
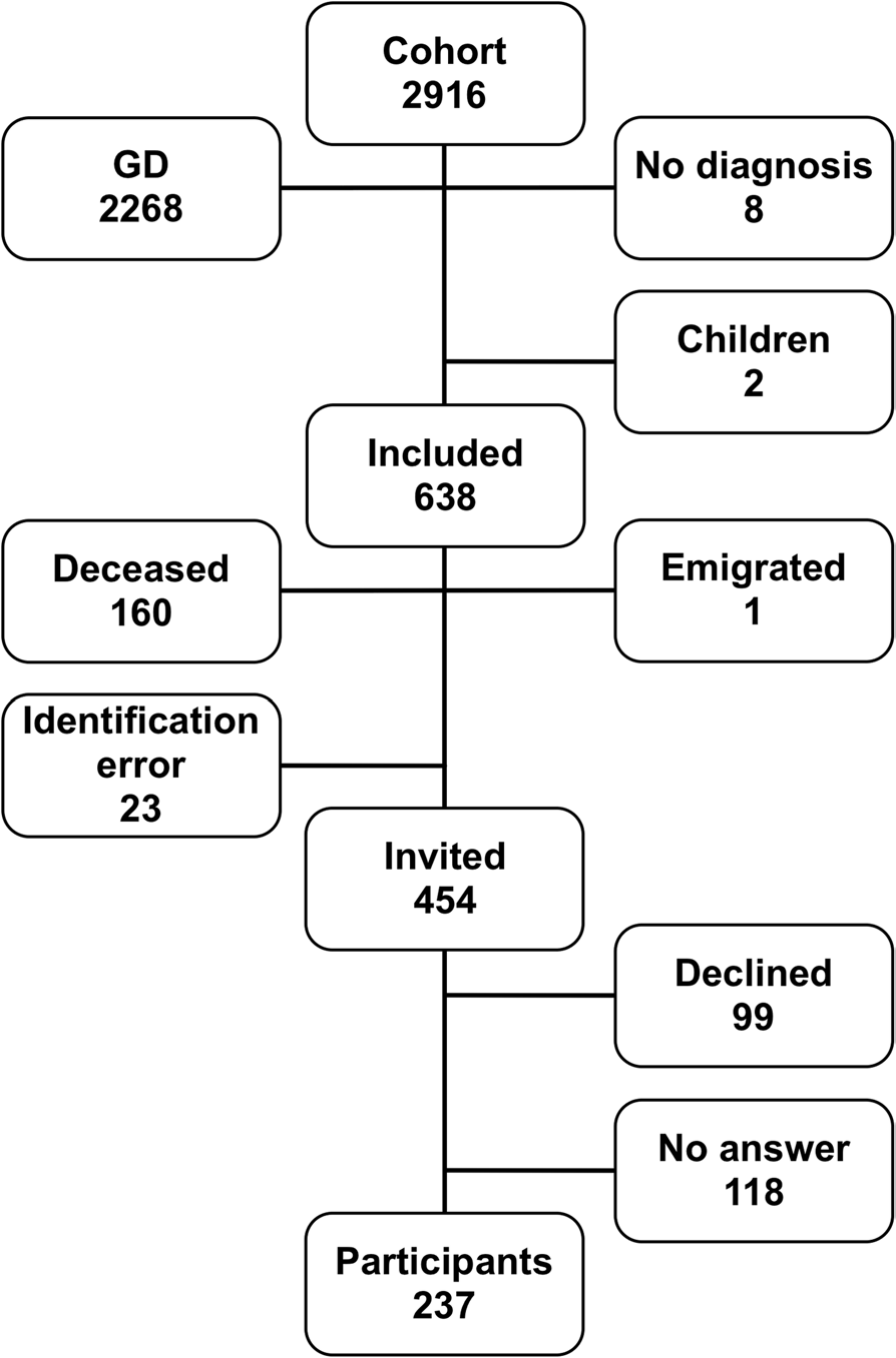
Flowchart illustrating the inclusion/exclusion of individuals in the study

In 2003–05, all patients with de novo hyperthyroidism (*n* = 2,916) were registered for an incidence study at thirteen endocrine clinics in seven Swedish cities covering 40% of the 9 million inhabitants in Sweden [1]. This is a follow-up study, six to ten years after the diagnosis, with a mean follow-up of 8 years (standard deviation 0.9) [20]. Of the 638 adult TNG patients present in the original cohort, 454 patients still alive were invited to participate in this study by mail (Fig. 1). If no reply was received, two postal reminders were sent, and finally, the patients were contacted by phone. With a response rate of 52%, 237 adult patients (above 18 years of age) agreed to participate, while 99 patients actively declined the study. The remaining 118 (26%) of the 454 invited patients did not reply and were defined as nonrepliers (Table 1).

**Table 1:** Baseline Data at Inclusion in Original Cohort 2003–2005 for the 237 Included Participants with Toxic Nodular Goiter in the Follow-Up 2011–2013 and for the 118 Individuals Who Did Not Reply to the Study Invitation (Nonrepliers)

		Included	Non-repliers	
		(*n* = 237)	(*n* = 118)	p
Gender				
	Women, n (%)	205 (86.5)	99 (83.9)	0.511
	Men, n (%)	32 (13.5)	19 (16.1)	
Age				
	Total mean years [SD]	62.1 [11.7]	60 [16.3]	0.170
	Women mean years [SD]	61.6[11.7]	59.7[16.9]	0.251
	Men mean years [SD]	65.1[11.2]	61.6[13.6]	0.325
Country of origin		(*n* = 237)	(*n* = 70)	
	Sweden, n (%)	202 (85.2)	55 (78.6)	0.185
	Europe outside Sweden, n (%)	22 (9.3)	5 (7.1)	0.579
	Outside Europe, n (%)	13 (5.5)	10 (14.3)	0.014
Smoking at diagnosis		(*n* = 234)	(*n* = 106)	
	Non-smoker, n (%)	91 (38.9)	46 (43.4)	0.433
	Former smoker, n (%)	72 (30.8)	32 (30.2)	0.914
	Smoking at diagnosis, n (%)	71 (30.3)	28 (26.4)	0.460
Eye symptoms		(*n* = 237)	(*n* = 117)	
	No Eye symptoms, n (%)	235 (99.2)	116 (99.1)	0.992
	Eye symptoms, n (%)	2 (0.8)	1 (0.9)	
Initial treatment		(*n* = 237)	(*n* = 53)	
	ATD, n (%)	40 (16.9)	17 (32.1)	0.012
	RAI, n (%)	167 (70.5)	33 (62.3)	0.243
	Surgery, n (%)	14 (5.9)	3 (5.7)	0.945
	Conservative, n (%)	16 (6.8)	0	0.052

The number of patients included in each analysis noted

*ATD* Antithyroid drugs, *RAI* Radioactive iodine, *SD* Standard deviation

To explore any possible sampling bias, the medical records of the 118 patients in the nonreplier group were reviewed by the authors with respect to treatment modality, order of treatments and recurrence of disease after each treatment course. For consistency, the same variables were checked in the 237 included patients´ medical files. Since no contact was established with the nonrepliers, QoL data could not be obtained. Of the 237 included patients, 170 (72%) returned the ThyPRO questionnaire. All treatment data was collected from medical journals to counter any recollection bias.

The TNG diagnosis included both toxic multinodular goitre and solitary toxic adenoma [1]. Patients diagnosed with TNG in the incidence study [1], where later data showed a positive TRAb, were reassigned to a GD diagnosis and excluded [20]. Patients with subclinical hyperthyroidism defined as only a low or suppressed thyroid-stimulating hormone were excluded [1].

### General treatment strategies for TNG in Sweden 2003–05

RAI was given to achieve euthyroidism with one treatment. The RAI dose was calculated for the patient to obtain 120–300 Gy, except in two centers (with 5.2% of the patients receiving RAI) where RAI was given as a fixed activity of 550 Mbq.

Lobectomy or total thyroidectomy were the most common surgical methods, depending upon the size and appearance of the thyroid on scintigraphy.

In cases where RAI and surgery cannot be performed, life-long ATD treatment is a second-line option. ATD was mostly administered as a block and replace treatment. Patients with mild disease were treated with beta-blockers only or were monitored with control of laboratory parameters (conservative treatment).

### Questionnaires

Patients were administered two questionnaires. A self-designed, clinical 68-item study-specific questionnaire, which, among many things, included demographic, life style and health questions. [20]. Second, the validated [21] thyroid-related QoL questionnaire ThyPRO measures thyroid-related QoL in terms of physical and mental symptoms, impaired function, well-being, and participation and has a negative impact on overall QoL [22]. In this study, a validated Swedish version of ThyPRO was used. In addition, a composite score summarizing the nine function, well-being, and participation scales can be achieved [23]. Each item is rated on a five-point Likert scale, and items are summarized and linearly transformed to 0–100 scales, with higher scores indicating more symptoms or impact. Reference material for the nine main categories of ThyPRO has been established based on the Danish general population [24].

### Data management and validation

All data sheets in the questionnaires were autoscanned and transferred into a database using licensed scanning software (Remark Office OMR 8©, Remark, Malvern, PA 19,355–1245 USA). The autoscanning was validated with manual control. Errors were observed in 0.92%, and those found were corrected. The database was also validated with a cross-check of medical records from 5% of randomly selected patients.

### Statistical analyses

The demographic data were analyzed using Pearson's chi-square or Fisher exact test for categorical variables and unpaired t-test for continuous variables. Statistical analyses were performed using IBM SPSS Statistics 22.0.0.1 64-bit edition (SPSS Institute, Chicago, IL, USA). Statistical significance was set at *p* < 0.05.

QoL was analyzed using SAS v9.4 (SAS Institute, Cary, NC). Differences in baseline characteristics and mean scale scores between the general Danish population and the TNG cohort, as well as among the three treatment groups, were evaluated with multiple linear regression using SAS PROC GLM. All estimations and tests were adjusted for age and sex.

### Ethics

This study was approved by the Regional Ethics Committee in Uppsala (Dnr 2012/035, April 4, 2012). The same committee additionally approved the review of the nonreplier group (Dnr 2012/035/2, Feb 24, 2015). The study was performed according to the Declaration of Helsinki.

## Results

### Baseline characteristics at diagnosis

The mean age was 62.1 years, and the female to male ratio was 6.4:1 (Table 1). The proportion of active smokers was 30.3%. In line with previous research, only two patients (0.8%) reported eye symptoms. The majority (85.2%) were born in Sweden. The TNG cohort was composed of 127 (53.6%) singular solitary toxic adenoma and 110 (46.4%) toxic multinodular goitre.

### Comparison to the nonreplier group

The included patients were more often born in Sweden and Europe and treated with RAI compared to the nonreplier group (Table 1).

### Long-term treatment outcome in TNG patients

**Fig. 2:**
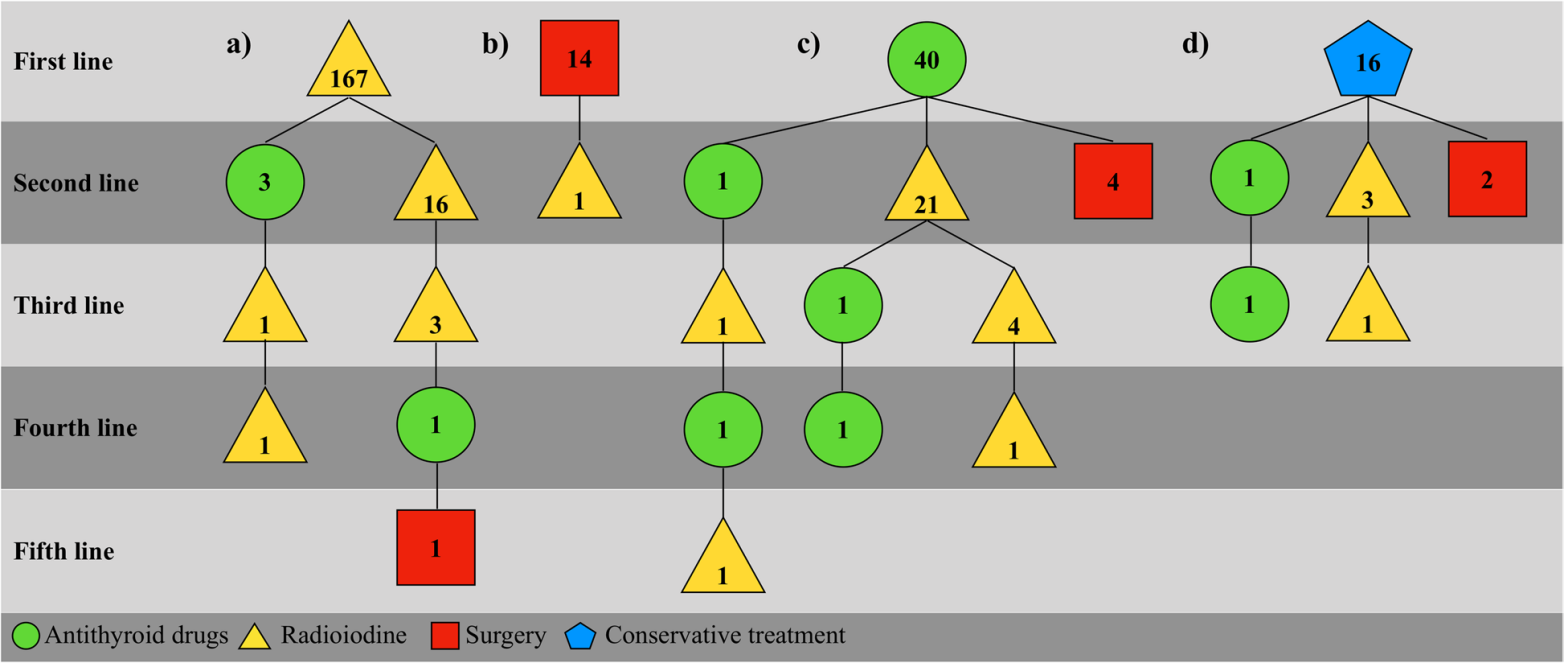
**a-d** Flowcharts illustrating the patients with toxic nodular goiter who initially received **a**) radioactive iodine, **b**) surgery, **c**) antithyroid drug treatment or **d**) conservative treatment and their subsequent treatment choices following change of previous treatment

RAI was used as first-line treatment in 167 (70.5%) patients, and 88.6% of these patients became euthyroid or hypothyroid. The remaining patients received additional RAI (*n* = 16) or ATD (*n* = 3) treatment. The second RAI course resulted in 13 (81.3%) patients becoming euthyroid or hypothyroid, and some patients needed up to three (five in total) additional treatments (Fig. 2a). Among those primarily treated with RAI, 96 (57.5%) were receiving levothyroxine by the end of the study period.

Surgery was used as first-line treatment in 14 patients (5.9%), and with this modality, 13 (92.9%) patients became euthyroid or hypothyroid. The remaining patient became euthyroid or hypothyroid after receiving RAI (Fig. 2b). Of those primarily treated with surgery, 9 (64.3%) were receiving levothyroxine by the end of the study period.

ATD was given in 16.9% (40/237) of the patients. Treatment failure or recurrent disease occurred in 26/40 (65.0%) of the ATD-treated patients (Fig. 2c). By the end of the study period, 25 (62.5%) patients continually used levothyroxine. The main part of the hypothyroid patients had also received RAI or surgery. Only 3 of the 14 patients who only received ATD used levothyroxine at follow-up.

Sixteen patients (6.8%) were treated conservatively (Fig. 2d). Of these patients, ten did not need further treatments.

In the whole group, five patients had a 4th treatment period, and two had a 5th (Fig. 2) of various treatment modalities.

### The patients’ evaluation of treatment outcome

At the end of the study period, 18.1% of the patients reported that they had not yet fully recovered. This answer was more common, but not significantly different, among those treated with RAI (21.1%) compared to ATD (12.8%) and surgery (8.3%). There were no significant differences in levothyroxine usage between the groups (data not shown).

### Quality of life

Patients with TNG had comparatively worse thyroid-related QoL in a broad spectrum than the Danish general population, as seen in Table 2. They differed in all nine scales, with the exception of the following three: Tiredness, Cognitive Complaints and Emotional Susceptibility. The most pronounced difference was in Eye Symptoms (ThyPRO-scale), followed by Hyperthyroid Symptoms, Anxiety and Depressivity. Small differences were also seen in Goitre Symptoms and Hypothyroid Symptoms.

**Table 2:** Mean (SD) ThyPRO scale scores for patients with toxic nodular goiter and the general reference population. Scale range 0–100, worse symptoms / more impact with increasing score

Scale	Toxic nodular goitre (*n* = 143–170)	Danish general population (*n* = 707–712)	Difference (95%CI)
Goiter Symptoms	9 (13)	5 (9)	4 (2 to 6)^a^
Hyperthyroid Symptoms	17 (16)	11 (13)	6 (3 to 8) ^a^
Hypothyroid Symptoms	18 (18)	14 (15)	4 (2 to 8) ^a^
Eye Symptoms	16 (17)	8 (10)	8 (7 to 11) ^a^
Tiredness	37 (25)	35 (21)	2 (-2 to 6)
Cognitive Complaints	16 (20)	14 (17)	2 (-1 to 6)
Anxiety	19 (25)	13 (16)	6 (3 to 11) ^a^
Depressivity	26 (21)	21 (18)	5 (1 to 8) ^a^
Emotional Susceptibility	21 (20)	23 (19)	2 (-1 to 6)
Impaired Social Life	7 (14)		
Impaired Daily Life	16 (24)		
Appearance	24 (32)		
Overall QoL Impact scale	8 (14)		
Composite scale	15 (27)		

^a^ Significant difference

There were no significant differences between the different treatments (data not shown).

## Discussion

In this study, we showed that TNG has long-term health effects, including impaired QoL and, for the majority of patients, a need for permanent levothyroxine therapy. Although the proportion of hypothyroidism is high, there was no significant difference between RAI and surgery, thus suggesting that one of the advantages of treating TNG with RAI compared to surgery is lost.. Patients with TNG had a lower QoL than the general population, but no reliable conclusions regarding differences between treatments could be made. Some patients requiring repeated treatments suggest a more complex course of TNG than expected. This, together with the long-term QoL impairments, is a reminder of the chronic nature of hyperthyroidism from TNG.

The rate of becoming euthyroid or hypothyroid with RAI as the first treatment was 89%, which is in accordance with previous data where retreatment with RAI was necessary in 10–30% of patients with a solitary toxic adenoma [7] and in 6–19% of patients with TNG [11, 25]. Patients who received a second RAI treatment became euthyroid or hypothyroid in 81% of cases. Five percent of TNG patients needed three or more treatments to become euthyroid or hypothyroid. Almost 60% of our RAI-treated patients were levothyroxine substituted at follow-up, which stands in contrast to the reports of hypothyroidism of between 0–36% in studies from the 1990s, although the RAI doses have decreased over time [6–8]. The length of these follow-up studies varies, but several are up to ten years; however, the number of patients in most articles is relatively small (fewer than 50 patients). A more recent study, on the other hand, seemed to indicate an even higher degree (73%) of hypothyroidism, and our findings are more in line with these results [9]. This can be due to a change in treatment, as RAI uptake tests are conducted more frequently today, together with a change in the attitude toward RAI, as hypothyroidism is not seen as an undesirable effect but as a result of a "quick cure."

With surgery, 93% of patients became euthyroid or hypothyroid, which is in line with previous reports [8] and did not, in comparison with RAI, result in significantly more patients ending up needing levothyroxine treatment at the end of the follow-up. Type of surgery is not recorded in the database, but this relatively low number of hypothyroid patients after surgery suggests a corresponding rate of lobectomy.

Previous studies show a seemingly large difference between RAI and surgery regarding hypothyroidism [6–8, 25], with a frequency of hypothyroidism after surgery of more than 80% [6, 8], which is significantly higher than that in our study. Thus, with no significant difference in the frequency of hypothyroidism, one of the benefits of treating TNG with RAI disappears in comparison to surgery. What remains are the risks of surgical complications, which is still a concern in thyroid surgery [6, 8].

ATD is not considered a primary treatment option in TNG due to the high prevalence of recurrent disease. Our data support this by showing that 17% of the patients received ATD as primary treatment, and only 35% of these patients became euthyroid or hypothyroid. The relatively high (63%) rate of substitution treatment of these patients can be attributed to the patients also receiving RAI or surgery. Almost 1/5 of the patients reported that they had not fully recovered at the end of the study period. As we know from detailed studies of these answers in a previous study with GD patients [20], the feeling of recovery depends on a variety of reasons, but it shows that these patients have symptoms or problems they relate to their hyperthyroid disease 8 years after diagnosis. In a previous study, levothyroxine usage accounted for a large proportion of these answers. In this study, however, there were no significant differences in levothyroxine usage between different treatments, and the results between treatments varied greatly, which might suggest that levothyroxine treatment does not affect QoL in patients treated for TNG.

Although QoL is low for TNG patients, previous studies have shown that it improves over time [17, 18]. It is mostly reduced before treatment and improves immediately after successful treatment [18, 19]. Six months after diagnosis, QoL has further improved, which indicates an improvement over time and that the reduction in QoL is temporary [18]. The current study shows, in contrast to previous studies, that the deterioration of QoL remains even after a longer period of time. As the same scale was used, it is within reason to compare our results, after 8 years, with the QoL at diagnosis and after 6 months from previous studies. The QoL shows a clear improvement after 8 years in comparison with patients with TNG before treatment and largely unchanged results in relation to the 6-month follow-up [18]. The difference between 8 years and 6 months is reduced tiredness, but anxiety and eye symptoms increase [18]. The latter could be attributed to the exclusion of patients with eye symptoms in the previous Danish study. However, in the current study, only two patients had eye symptoms, so this result is reasonable not because of the difference in exclusion criteria between the studies. The question remains as to what causes this reduction in QoL.

One hypothesis is that hyperthyroidism on its own affects QoL, which may be supported by several studies showing a decrease in QoL when comparing a general population not only to TNG but also to GD and hyperthyroidism regardless of the cause [16–18]. Regardless of the cause, the same pattern of QoL improvement after treatment and over time was seen. Arguments against this theory might be that patients with nontoxic goitre also have a decreased QoL, which also seems to persist over time and after treatment [24, 26]. In a previous study [27], with patients from the same database, the QoL for GD patients was assessed. Comparing these results of QoL in GD patients with our TNG patients, TNG has a generally higher QoL than GD treated with RAI after 8 years. This also applies to the other treatments (ATD and surgery), with the exception that TNG patients assess their feeling of depression as worse. No differences can be seen between the treatment groups in this analysis, which could mean that there is no difference, or it could be due to the number of patients treated with ATD and surgery being so small, with too low power for calculations.

The quality of life for the TNG group may have been overestimated as a result of the mortality rate in this follow-up study.

In comparison to the patients who were included, the non-responder group received ATD treatments more frequently. Since no patients in the non-responder group received conservative care, this is somewhat mitigated. The main limitation of this study is the lack of QoL data at baseline. Additionally, the general population sample was from Denmark, and the studied patients were from Sweden. There could be a difference in QoL between the countries that influences the outcomes. Furthermore, due to low power concerning the treatment groups, no conclusions regarding the difference in QoL between treatments can be made. Other limitations include the lack of duration of treatment and sufficient data regarding laboratory measurements. The type of surgery is not recorded in the database, making the relevance of the difference in hypothyroidism between patients treated with RAI vs surgery harder to interpret.

The response rate was only 52%, although this is not unusual in follow-up studies of this size and duration. One reason for this might be a high mean age of approximately 70 years old at follow-up. Even though this is among the largest studies concerning TNG, the treatment subgroups are very small.

There are a number of strengths of our study. The descriptive study design reflects our usual practice, all three treatment modalities are included, the cohort is large compared to most studies of TNG, and the long follow-up of a predefined incidence cohort limits selection bias. An additional strength is our investigation of the nonreplier group comprising 26% (118/454) of the total initial cohort in addressing possible major collection bias. TNG is also considerably less explored than GD, and it is thus important to conduct more research in patients with TNG regarding QoL and treatment outcomes.

## Data Availability

Due to the nature of this research, participants of this study did not agree for their data to be shared publicly, so supporting data is not available.

## Abbreviations

ATD: Antithyroid drugs
GD: Graves’ disease
QoL: Quality of life
RAI: Radioactive iodine
TNG: Toxic nodular goitre
